# Prevalence and factors of discordance attitudes toward advance care planning between older patients and their family members in the primary medical and healthcare institution

**DOI:** 10.3389/fpubh.2023.1013719

**Published:** 2023-02-23

**Authors:** Ling Ye, Genhong Jin, Min Chen, Xingyuan Xie, Shanshan Shen, Song Qiao

**Affiliations:** ^1^Department of Geriatrics, Jinhua Fifth Hospital, Jinhua, China; ^2^Department of Geriatrics, Zhejiang Hospital, Hangzhou, China; ^3^Department of Neurology, Zhejiang Hospital, Hangzhou, China

**Keywords:** advance care planning, attitudes, older adults, geriatric assessment, advance directives

## Abstract

**Background:**

This study aimed at investigating the prevalence and factors of the discordant attitudes toward advance care planning (ACP) among older patients and their family members toward patients' engagement in ACP in the primary medical and healthcare institution.

**Methods:**

In a cross-sectional study, a total of 117 older patients and 117 family members from Jinhua Fifth Hospital in China were enrolled. The questionnaire included sociodemographic characteristics, functional capacity assessment, and attitudes toward patients' engagement in ACP. Functional capacity assessment scales included the Modified Barthel Index (MBI), the Short-Form Mini-Nutritional Assessment (MNA-SF), the 15-item Geriatric Depression Scale (GDS-15), the Mini-Mental State Examination (MMSE), the Clinical Frailty Scale (CFS), and the SARC-F questionnaire.

**Results:**

The discordance attitudes toward patients' engagement in ACP between patients and family members accounted for 41(35.0%). In the multivariate logistic analysis, factors associated with higher odds of discordance attitudes toward patients' engagement in ACP included greater age differences between patients and family members (*OR* = 1.043, 95% *CI*: 1.007–1.081), lower educational level for family members (*OR* = 3.373, 95% *CI*: 1.239–9.181), the patient's higher GDS-15 score (*OR* = 1.437, 95% *CI*: 1.185–1.742), and patient's higher MNA-SF score (*OR* = 1.754, 95% *CI*: 1.316–2.338).

**Conclusion:**

Older patients and their family members had little ACP knowledge, and factors that influence discordance attitudes toward patients' engagement in ACP included the age gaps between patients and family members, family members' educational level, patients' depressive symptoms, and patients' nutritional status.

## 1. Introduction

The acceleration of population aging in China has brought more challenges to elderly care services, medical and social resources. And end-of-life care issues such as living wills and place of death have become a problem that cannot be ignored. In order to promote death with dignity, it is an urgent task to build a healthy China under the current aging situation. Therefore, the Healthy China 2030 Plan and other far-reaching reforms released by the Chinese Government have pointed out the necessity of palliative care and hospice care including the implementation of advance care planning (ACP). ACP refers to a process wherein individuals with clearly aware decision-making capacity reflect upon personal life experience and values to make their future care goals and treatment preferences in advance (1, 2). As an important concept of ACP, advance directives (ADs) are usually in a legal formal written document that nominates a substitute decision proxy and/or determined life-sustaining treatment through a living will (3). The implementation of ACP is associated with realizing medical autonomy (3), relieving stress, anxiety, and depression in family members (4), reducing over-utilization of aggressive measures during the end of life (5–7), cutting down Medicare costs (8), and decreasing in-hospital mortality (9, 10).

Research on ACP for older adults in China is still in the early stage, and only one city on the mainland currently has such relevant local legal regulations. Of cognitively normal Chinese older adults from 140 nursing homes in Hong Kong, 88% of older residents preferred having ADs regarding their future medical treatments (11). A multicenter cross-sectional study from 25 hospitals throughout mainland China included 91.1% of older patients aged over 60 years, and the results reported that 38.3% of patients had heard about ACP, and 50.6% were willing to carry out ACP when being informed about relevant knowledge of ACP (12). The study clarified attitudes and preferences toward ACP in a relatively Chinese large sample mostly in tertiary hospitals but did not involve the elderly population in the primary medical and health care institution and the influence of functional status on ACP attitudes. Prior research has compared attitudes toward ACP between patients and family members, which has focused on specific diseases such as cancer and heart failure (13, 14). Different from the other specialized wards with a certain specific disease, older patients in the geriatric wards in the primary medical and health care institution, may have advanced age, complex multimorbidity, multiple functional loss, and a higher proportion at the final stage of the disease. It may be more common to hide the true condition of the patients and make medical decisions on behalf of their family members. Prior studies have suggested that despite their family members understanding the patient's wishes regarding end-of-life care, frequent disagreement between them about treatment preferences and goals of care often arises (15). However, medical decisions that are in concordance with seriously ill patients' values and goals are regarded as high-quality care (16, 17). Discordance with the patient's values, goals, and medical treatments has been shown to increase medical costs and prolong end-of-life difficulties (17–19). Additionally, other factors including health status, family support, physical functioning, and experiences of family or relatives rescuing may affect their perceptions of ACP and end-of-life care.

Thus, this study aimed at investigating the attitudes and preferences of older patients and their family members toward patients' engagement in ACP in a primary medical and healthcare institution. Moreover, this study integrated factors such as the functional capacity to explore the associated factors on discordance attitudes toward ACP between patients and their family members.

## 2. Methods

### 2.1. Study design and setting

A cross-sectional study was carried out in the Department of Geriatrics in Jinhua Fifth Hospital between October 2020 and August 2021. Jinhua Fifth Hospital, the first filed public hospital for old-age care in Jinhua City, Zhejiang Province, mainly serves the elderly with disability and multiple comorbidities. The medical and old-age care integration model in the primary medical and health care institution refers to integrating medical care, rehabilitation, nursing, and life care, and is an effective means to improve the quality of old-age care.

### 2.2. Participants

One hundred seventeen patients and 117 family members from Jinhua Fifth Hospital were enrolled by convenience sampling. Patients' inclusion criteria: (a) Age ≥ 60 years; (b) Patients with clear consciousness who have no communication barriers; (c) Patients who can sign informed consent voluntarily and cooperate in completing the investigation. Patients' exclusion criteria: unable to cooperate to complete the ACP questionnaire because of consciousness disorder, severe cognitive impairment, and other critical conditions. Family members' inclusion criteria: family members of hospitalized patients who voluntarily participated and were able to cooperate in completing the study.

This study was approved by the Medical Ethics Committee of Jinhua Fifth Hospital (number: 2021-04), and written informed consent was obtained from the patients and their family members prior to the data collection.

### 2.3. Measurements

Both patients and family members completed the questionnaires about sociodemographic and ACP attitudes, and functional capacity assessment by comprehensive geriatric assessment (CGA) was only investigated by the patients. Patients and family members separately expressed their own perspectives on ACP through face-to-face interviews.

Sociodemographic data including age, sex, marital status (categorized by married, divorced, widow, or single), educational level (classified as high school or below), medical insurance, religion, the relationship between patients and caregivers, self-reported family support (coded as poor, fair, and good), self-reported health status (coded as poor, fair, and good), concurrent diseases (including coronary artery disease, hypertension, diabetes, cerebrovascular disease, respiratory disease, and osteoarticular diseases), and prescription medications were recorded.

The functional capacity assessment was conducted by CGA based on the Chinese expert consensus recommendation (20). In this study, the activity of daily living was assessed by the Modified Barthel Index (MBI), and the higher the MBI score indicated the better the activity of daily living (21). The Short-Form Mini-Nutritional Assessment (MNA-SF) was used to ascertain the degree of malnutrition risk (22). Depressive symptoms were evaluated using the 15-item Geriatric Depression Scale (GDS-15), with higher scores indicating more depressive symptoms (23). Cognitive function was assessed using the Mini-Mental State Examination (MMSE) (24). Higher MMSE score indicated better cognitive function. Frailty was detected by the Clinical Frailty Scale (CFS) which was scored from 1 (very fit) to 9 (severely frail) (25). Based on the clinical judgment, a higher CFS score was considered a higher degree of frailty. The SARC-F questionnaire was used to screen sarcopenia, with higher values indicating a greater likelihood of sarcopenia (26).

A structured questionnaire about ACP attitudes was completed independently by patients and their family members. The questionnaire included prior experience with relatives and friends being rescued (coded as yes or no), attitudes toward death (categorized by fear, avoid discussing, and accept discussing), ACP knowledge, determination surrogate, value statement about end-of-life (coded as active treatment, relieving uncomfortable symptoms, maintenance of daily function, and quality of life or unknown), preferences for end-of-life treatments (including cardiopulmonary resuscitation, invasive mechanical ventilation support, non-invasive ventilation support, renal replacement therapy, gastrointestinal colostomy, nasal tube, deep vein catheterization, urinary catheter, and transfusion), and desired place of death. Discordance attitudes were defined based on patients' and family members' responses to the question about whether to consider ACP engagement of patients if patients cannot make decisions due to a medical condition (such as coma).

### 2.4. Data collection process

Patients and their family members were informed of the aim and the detailed process of the study when they visited the Department of Geriatrics. After obtaining their informed consent, they were interviewed by a trained researcher and the data were analyzed by another researcher.

### 2.5. Sample size calculation

A sample size of 111 patients was calculated to detect a discordance rate (p) of 32% according to a previous study (27), assuming a type I error (α) of 0.05, a desired precision (d) was 0.05, and a two-sided test. N represents the estimated annual cases of 165 new elderly patients admitted to the geriatrics department of the primary medical and healthcare institution. A non-response rate was set as 5%, and 117 pairs of patients and family members were required. The formula is as follows:


(1) $$\begin{array}{l} n={\frac{{\left(\frac{Z_{\alpha }}{\delta }\right)}^{2}*p*\left(1-p\right)}{1+\left[{\left(\frac{Z_{\alpha }}{\delta }\right)}^{2}*p*\left(1-p\right)\right]/N}}^{} \end{array}$$

### 2.6. Statistical analysis

Data were analyzed using SPSS 18.0 software (SPSS, Chicago, IL, USA). The frequency and distribution tested by the normality test for all variables were evaluated. The continuous variables included patients' age, family members' age, the age gap between patients and family members, and functional capacities. These variables were presented as median (interquartile range, IQR) because they were not normally distributed, and the Mann-Whitney U tests were used to compare the differences between groups. Theχ^2^ tests were used to estimate differences in other variables between groups, and dichotomous variables are expressed as numbers (percentages). Furthermore, a multivariate logistic regression model to estimate odds ratios (*OR*s) and 95% confidence intervals (*CI*s) was conducted to identify associated influencing variables with discordance attitudes toward ACP between older patients and their family members. The variables with *P* < 0.2 in bivariate analysis were selected in the multivariate logistic regression analysis. A *P*-value of < 0.05 was considered statistical significance.

## 3. Results

Table 1 presents the characteristics of 117 eligible pairs of patients and family members. Among patients, 66 (56.4%) were male, with a median age of 80 years. Among family members, 52 (44.4%) were males, with a median age of 60 years. Significant differences were found in age, marital status, educational level, self-reported health status, and comorbid diseases between patients and family members (all *P* < 0.05).

**Table 1:** Characteristics of older patients and their family members.

**Variables**	**Patients (*n* = 117)**	**Family members** **(*n* = 117)**	***Z* or *X^2^***	***P*-value**
Age, median (IQR), scores^#^	80.0 (69.5, 87.0)	60.0 (52.0, 65.0)	−10.032	<0.001
Male, *n* (%)	66 (56.4)	52 (44.4)	3.351	0.067
Married, *n* (%)	87 (74.4)	110 (94.0)	16.983	<0.001
High school or above, *n* (%)	30 (25.6)	70 (59.8)	27.940	<0.001
Self-reported health status, *n* (%)			86.576	<0.001
Poor/fair	98 (83.8)	27 (23.1)		
Good	19 (16.2)	90 (76.9)		
**Comorbidities**, ***n*** **(%)**				
Coronary artery disease	30 (25.6)	3 (2.6)	25.718	<0.001
Hypertension	57 (48.7)	7 (6.0)	53.768	<0.001
Diabetes	26 (22.2)	1 (0.9)	26.167	<0.001
Cerebrovascular disease	15 (12.8)	0 (0)	16.027	<0.001
Respiratory disease	26 (22.2)	1 (0.9)	26.167	<0.001
Osteoarticular diseases	18 (15.4)	1 (0.9)	16.555	<0.001
**Caregiver relationship**, ***n*** **(%)**				
Spouses	NA	29 (24.8)		
Children	NA	81 (69.2)		
Sibling/relatives	NA	7 (6.0)		
**Self-reported family support**, ***n*** **(%)**				
Poor/fair	41 (35.0)	NA		
Good	76 (65.0)	NA		
MMSE, median (IQR), scores^#^	19.0 (15.0, 25.0)	NA		
GDS-15, median (IQR), scores^#^	7.0 (5.0, 8.0)	NA		
MBI, median (IQR), scores^#^	90.0 (75.0, 97.5)	NA		
MNA-SF, median (IQR), scores^#^	12.0 (9.0, 12.0)	NA		
CFS, median (IQR), scores^#^	5.0 (3.0, 6.0)	NA		
SARC-F, median (IQR), scores^#^	3.0 (0, 4.0)	NA		

IQR, interquartile range; MMSE, the Mini-Mental State Examination; GDS-15, the 15 item Geriatric Depression Scale; MBI, the Modified Barthel Index; MNA-SF, the Short-Form Mini-Nutritional Assessment; CFS, the Clinical Frailty Scale.

^#^The Mann-Whitney U tests.

Table 2 displays the attitudes of older patients and their family members toward ACP and end-of-life treatments for the patients. About 84% of patients chose their children as their medical decision-making surrogates. Family members were more willing to actively discuss death with patients in order to cope with the subsequent irreversible final stage of life, but, in fact, family members preferred to choose active treatment for patients. In regards to the preferred place of death, there was no significance between patients and family members.

**Table 2:** Comparison of attitudes of older patients and their family members toward ACP and end-of-life treatments for the patients.

**Variables**	**Patients (*n* = 117)**	**Family members (*n* = 117)**	***X^2^***	***P*-value**
Surrogate, *n* (%)		NA		
Self	10 (8.5)			
Spouses	8 (6.8)			
Children	98 (83.8)			
Sibling/relatives	1 (0.9)			
Prior experience relatives and friends being rescued, *n* (%)	63 (53.8)	57 (48.7)	0.616	0.433
Attitude toward death, *n* (%)			7.672	0.022
Fear	14 (12.0)	6 (5.1)		
Avoid discussing	24 (20.5)	14 (12.0)		
Accept discussing	79 (67.5)	97 (82.9)		
Value statement about end-of-life care, *n* (%)			55.658	<0.001
Active treatment	31 (26.5)	88 (75.2)		
Relieve uncomfortable symptoms	67 (57.3)	23 (19.7)		
Maintenance daily function and quality of life	8 (6.8)	3 (2.6)		
Unknown	11 (9.4)	3 (2.6)		
Preferences for end-of-life treatments				
Cardiopulmonary resuscitation, *n* (%)			25.435	<0.001
Yes	53 (45.3)	88 (75.2)		
No	21 (17.9)	16 (13.7)		
Unknown	43 (36.8)	13 (11.1)		
Invasive mechanical ventilation support, *n* (%)			19.549	<0.001
Yes	27 (23.1)	36 (30.8)		
No	47 (40.2)	67 (57.3)		
Unknown	43 (36.8)	14 (12.0)		
Non-invasive ventilation support, *n* (%)			26.684	<0.001
Yes	52 (44.4)	89 (76.1)		
No	23 (19.7)	15 (12.8)		
Unknown	42 (35.9)	13 (11.1)		
Renal replacement therapy, *n* (%)			27.492	<0.001
Yes	42 (35.9)	78 (66.7)		
No	32 (27.4)	26 (22.2)		
Unknown	43 (36.8)	13 (11.1)		
Gastrointestinal colostomy, *n* (%)			23.793	<0.001
Yes	43 (36.8)	77 (65.8)		
No	31 (26.5)	25 (21.4)		
Unknown	43 (36.8)	15 (12.8)		
Nasal tube, *n* (%)			29.222	<0.001
Yes	48 (41.0)	87 (74.4)		
No	26 (22.2)	17 (14.5)		
Unknown	43 (36.8)	13 (11.1)		
Deep vein catheterization, *n* (%)			27.352	<0.001
Yes	49 (41.9)	86 (73.5)		
No	25 (21.4)	18 (15.4)		
Unknown	43 (36.8)	13 (11.1)		
Urinary catheter, *n* (%)			28.289	<0.001
Yes	51 (43.6)	89 (76.1)		
No	23 (19.7)	15 (12.8)		
Unknown	42 (35.9)	13 (11.1)		
Transfusion, *n* (%)			28.328	<0.001
Yes	50 (42.7)	89 (76.1)		
No	24 (20.5)	14 (12.0)		
Unknown	43 (36.8)	14 (12.0)		
Preferred place of death, *n* (%)			5.364	0.068
Home	20 (17.1)	13 (11.1)		
Medical or elderly care institutions	6 (5.1)	15 (12.8)		
General hospital	91 (77.8)	89 (76.1)		

Only 4 patients (3.4%) and 14 family members (12.0%) heard of ACP. When the ACP was fully informed, the percentages of instituting ACP in the irreversible final stage of life increased to 51.3 and 78.6%, respectively. However, the discordant attitudes toward ACP between patients and family members accounted for 41 (35.0%). In the bivariate analysis, several patients' and family members' factors were associated with discordant attitudes toward ACP (Table 3). In the multivariate logistic analysis, factors associated with higher odds of discordance attitudes toward ACP included greater age differences between patients and family members (*OR* = 1.043, 95% *CI*: 1.007–1.081), higher GDS-15 score (*OR* = 1.437, 95% *CI*: 1.185–1.742), higher MNA-SF score (*OR* = 1.754, 95% *CI*: 1.316–2.338), and lower educational level for family members (*OR* = 3.373, 95% *CI*: 1.239–9.181) (Table 4).

**Table 3:** The patient and family member factors associated with discordance attitudes toward ACP by bivariate analysis.

**Variables**	**Accordance (*n* = 76)**	**Discordance (*n* = 41)**	***Z* or *X^2^***	***P*-value**
**Patient**				
Age, median (IQR), years^#^	80.0 (70.0, 87.0)	79.0 (65.5, 86.5)	−0.223	0.824
Age difference between patient and caregiver, median (IQR), scores^#^	23.0 (3.0, 28.8)	26.0 (7.5, 32.0)	−1.872	0.061
Male, *n* (%)	45 (59.2)	21 (51.2)	0.692	0.406
Married, *n* (%)	53 (69.7)	34 (82.9)	2.430	0.119
High school or above, *n* (%)	20 (26.3)	10 (24.4)	0.052	0.820
Self-reported health status, *n* (%)			0.451	0.502
Poor/fair	55 (72.4)	32 (78.0)		
Good	21 (27.6)	9 (22.0)		
Self-reported family support, *n* (%)			6.688	0.010
Poor/Fair	33 (43.4)	8 (19.5)		
Good	42 (56.6)	33 (80.5)		
**Comorbidities**, ***n*** **(%)**				
Coronary artery disease	18 (23.7)	12 (29.3)	0.436	0.509
Hypertension	35 (46.1)	22 (53.7)	0.617	0.432
Diabetes	18 (23.7)	8 (19.5)	0.268	0.605
Cerebrovascular disease	11 (14.5)	4 (9.8)	0.530	0.466
Respiratory disease	20 (26.3)	6 (14.6)	2.103	0.147
Osteoarticular diseases	11 (14.5)	7 (15.4)	0.138	0.710
Prior experience relatives and friends rescued, *n* (%)	34 (44.7)	29 (70.7)	7.241	0.007
MMSE, median (IQR), scores^#^	21.0 (16.0, 25.0)	16.5 (14.0, 25.8)	−0.852	0.394
GDS-15, median (IQR), scores^#^	6.0 (3.0, 8.0)	7.5 (7.0, 8.0)	−3.596	<0.001
MBI, median (IQR), scores^#^	90.0 (70.0, 95.0)	90.0 (80.0, 100.0)	−1.125	0.261
MNA-SF, median (IQR), scores^#^	12.0 (8.0, 12.0)	12.0 (11.5, 12.0)	−2.621	0.009
CFS, median (IQR), scores^#^	5.0 (3.3, 6.0)	5.0 (3.0, 5.0)	−1.780	0.075
SARC-F, median (IQR), scores^#^	2.5 (0, 4.0)	3.0 (0, 4.0)	−0.268	0.789
**Family member**				
Age, median (IQR), scores^#^	60.0 (53.3, 65.0)	57.0 (49.5, 62.5)	−1.896	0.058
Male, *n* (%)	30 (39.5)	22 (53.7)	2.170	0.141
Married, *n* (%)	74 (97.4)	36 (87.8)		0.050
High school or above, *n* (%)	49 (64.5)	21 (51.2)	1.947	0.163
Self-reported health status, *n* (%)			1.282	0.258
Poor/fair	20 (26.3)	34 (82.9)		
Good	56 (73.7)	7 (17.1)		
Prior experience relatives and friends being rescued, *n* (%)	43 (56.6)	14 (34.1)	5.364	0.021

IQR, interquartile range; MMSE, the Mini-Mental State Examination; GDS-15, the 15 item Geriatric Depression Scale; MBI, the Modified Barthel Index; MNA-SF, the Short-Form Mini-Nutritional Assessment; CFS, the Clinical Frailty Scale.

^#^The Mann-Whitney U tests.

**Table 4:** The patient and family member factors associated with discordance attitudes toward ACP by multivariate logistic analysis.

	***OR* (95% *CI*)**	***P*-value**
**Patient**		
Age differences between patients and family members	1.043 (1.007, 1.081)	0.019
GDS-15 score	1.437 (1.185, 1.742)	<0.001
MNA-SF score	1.754 (1.316, 2.338)	<0.001
**Family member**		
High school or above	Ref	
Junior high and below	3.373 (1.239, 9.181)	0.017

MMSE, the Mini-Mental State Examination; GDS-15, the 15 item Geriatric Depression Scale; MBI, the Modified Barthel Index; MNA-SF, the Short-Form Mini-Nutritional Assessment; CFS, the Clinical Frailty Scale.

The model adjusted for age difference between patient and family member, patient covariates (marriage status, family support, health status, prior experience relatives and friends rescued, GDS-15 score, MNA-SF score, CFS score), and family member covariates (sex, marriage status, education level, prior experience relatives and friends rescued).

## 4. Discussion

This study included older patients and their family members in the primary medical and healthcare institution, and identified that discordance attitudes toward patients' engagement in ACP between them were common, with the discordance rate accounting for ~35%. More specifically, most patients and their family members viewed general hospitals as the preferred location of death, but family members would choose more aggressive life-sustaining treatments for patients at the end of life than patients themselves. Indeed, multiple previous studies demonstrated poor patient-surrogate agreement about patients' end-of-life treatment preferences (15, 27, 28). One study showed that agreement between older persons and their surrogates regarding living will completion were 81%, while agreement about the other aspects of ACP including healthcare surrogates, attitudes toward life-sustaining treatments, and the quality and quantity of life was 62–68% (27). The low compliance of patients' end-of-life preferences may be attributed to the lack of ACP knowledge. The dissemination and implementation of ACP need to take into account cultural and ethical considerations (29, 30). It is well known that people in a Western culture attach great importance to patient autonomy and quality of life, partly because they have received death education since childhood, as well as the legislative power of patient autonomy and informed consent (31, 32). However, adult children in Chinese traditional culture often act on the patient's preferred surrogates for future medical decisions, they are endowed with important family responsibilities to make every effort to prolong their older patients' lives. And the collectivism of family and society is considered as having a higher value than patient autonomy in end-of-care decision-making, which prevents ACP discussion by families who are reluctant to inform patients of their true condition and discuss death (33). In addition, ethical conflicts about what is a reasonable decision for a patient end of life care often occur during ACP communication and the decision-making process (34, 35).

In addition to cultural and ethical considerations, our study found that discordance attitudes varied greatly with respect to age gaps between patients and family members, family members' educational level, patients' depressive symptoms, and patients' nutritional status. The smaller the age gap between the patient and his family member is, that is, they are both in advanced age, the easier the family members understand the patient's preference. On the contrary, the greater the age gap between the two, the younger family members may make decisions against the patient's will due to traditional culture, ethics, and other factors. There are no relevant studies to explore the association between the age gap and disagreement attitudes toward ACP activities between patients and their family members. Thus, the result of this study needs to be further warranted in a large sample study. Moreover, the awareness rates of ACP knowledge in both older patients and their family members in this study were obviously lower than the previously reported rates in tertiary general hospitals (12), and it may be supportive of the importance of promoting ACP education in the primary institutions. Except for ACP education, original educational level is known to influence individuals' attitudes toward ACP, and our study revealed that poorly educated family members were more prone to make decisions that were against the patients' end-of-life preferences than those with highly educated. In accordance with a recent study investigating factors influencing older married couples possessing an AD, the result clarified that older couples in which one or both spouses went to college were more prone to report AD concordance (36). Compared with poorly educated family members, highly educated family members may have more access to increase their knowledge and understanding of patients' wishes, and are more likely to joint communicate end-of-life treatment and care preferences with their elders, thus reducing the burden of making difficult end-of-life decisions on behalf of patients.

Functional capacity parameters, especially depressive symptoms and the nutritional status of patients were identified as important associated modifiable factors. Evidence showed that depression was associated with enhancing discussions about end-of-life care and declining cardiopulmonary resuscitation (37, 38), and a decrease in depressive symptoms, in turn, increased the likelihood of patients changing preferences from declining to desiring cardiopulmonary resuscitation (39). ACP discussion and intervention could facilitate alleviating anxiety and depressive symptoms of terminally ill patients and their surviving relatives, but neither improves the quality of life nor the end-of-life care decision-making process (4, 40). Fluctuations in patients' depressive symptoms and lack of communication may increase the possibility of inconsistent attitudes toward ACP. Furthermore, the patient's poor nutritional status was associated with an accordance attitude toward ACP. The observed association can be explained by the fact that older patients with malnutrition were accompanied by multiple comorbidities, reduced physical functioning, and dependence on activities of daily living (41). Poor physical condition and dependence synergistically make older patients a more self-perceived burden to their families (42). Older adults often have a perception that they do not want to burden others, including their families (43, 44). Moreover, prior studies of the perspective of the elderly on ACP have shown that ACP would ease the family burden (45). Hence, increasing family burdens seems to be an important factor in end-of-life decision-making for older adults. The discordant attitudes toward ACP between older patients and their family members were seen in older patients with good nutritional status in this context.

Studies have shown ACP focused more on improved concordance of care, particularly at the end of life, rather than improved clinical outcomes (46). Another study described the ACP process as part of chronic disease management (47). Based on these findings, the integration of ACP for older patients in the primary medical and health care institution into routine care may facilitate informed and shared decision-making in regard to complicated therapeutic options and palliative care that is in line with personal values and preferences. This study identified several modifiable and non-modifiable factors toward ACP discordance attitudes, which were important for good communication between older patients and their family members. It is suggested that clinicians need to pay close attention to the potentially vulnerable groups with discordant attitudes, and patient-family-clinician shared decision-making about end-of-life preferences should be adopted to achieve the goal of honoring patients' values, preferences, and wishes. In addition to ACP education, the ACP-related laws and regulations, and the robust healthcare system need to be supported at the national level in order to implement ACP smoothly.

However, this study also has some limitations. Firstly, this study recorded older patients and their family members' perceived attitudes toward future ACP engagement of patients, rather than actual discordance in medical care and treatments received. Attitudes toward end-of-life preferences would change during hospitalization for some patients and their family members, due to various reasons. Secondly, this study did not explore physician preferences for the patient's care goals and treatments. Thirdly, this study was conducted in a single institution with a relatively small sample, and the data were collected at one point in time. Thus, the findings were of limited generality, and no causality could be assumed. Fourth, the lack of collection of response rates and characteristics of non-responders may result in biased prevalence estimates and selection bias, and the results should be interpreted with caution.

## 5. Conclusion

This study indicated that older patients and their family members had little ACP knowledge, and factors that influence discordance attitudes toward patients' engagement in ACP included age gaps between patients and family members, family members' educational level, patients' depressive symptoms, and patients' nutritional status. Early ACP education for older patients and their family members may promote ACP communications, and thus facilitate patient-family-clinician shared decision-making in the primary medical and healthcare institution, which eventually achieves the goal of honoring patients' values, preferences, and wishes.

## Data availability statement

The original contributions presented in the study are included in the article/supplementary material, further inquiries can be directed to the corresponding authors.

## Ethics statement

The studies involving human participants were reviewed and approved by the Medical Ethics Committee of Jinhua Fifth Hospital. The patients/participants provided their written informed consent to participate in this study.

## Author contributions

SS and SQ contributed to the conceptualization and methodology. LY and SS analyzed the data and wrote the original draft. LY, GJ, MC, and XX contributed to data collection. All authors contributed to implementing and revising the manuscript. All authors contributed to the article and approved the submitted version.
